# Safety of Magnetic Resonance Imaging in Patients with Cardiac Implantable Electronic Devices

**DOI:** 10.3390/jcdd11100313

**Published:** 2024-10-08

**Authors:** Hugo Lanz, Katharina Strauß, Julia Höpler, Marie Kraft, Sabine Hoffmann, Leonhard Binzenhöfer, Nils Gade, Daniel Roden, Inas Saleh, Stefan Kääb, Korbinian Lackermair, Sebastian Sadoni, Christian Hagl, Steffen Massberg, Heidi Estner, Stephanie Fichtner, Enzo Lüsebrink

**Affiliations:** 1 Department of Medicine I, LMU University Hospital, 81377 Munich, Germany; hugo.lanz@med.uni-muenchen.de (H.L.); katharina.strauss@med.uni-muenchen.de (K.S.); stephanie.fichtner@lakumed.de (S.F.); 2 DZHK (German Center for Cardiovascular Research), Partner Site Munich Heart Alliance, 80636 Munich, Germany; 3 Institute for Medical Information Processing, Biometry, and Epidemiology, Ludwig-Maximilians-Universität München, 81377 Munich, Germany; 4 Departments of Cardiac Surgery, LMU University Hospital, 81377 Munich, Germany

**Keywords:** magnetic resonance imaging, cardiac implantable electronic device, pacemaker, defibrillator

## Abstract

Background: MRI (magnetic resonance imaging) represents the diagnostic image modality of choice in several conditions. With an increasing number of patients requiring MRI for diagnostic purposes, the issue of safety in patients with cardiac implantable electronic devices (CIED) undergoing this imaging modality will play an ever more important role. The purpose of this study was to assess the safety and device function following MRI in an unrestricted real-world cohort of patients with a wide array of cardiac devices. Methods: We conducted a retrospective single-center study including 1010 MRI studies conducted in adult patients (≥18 years) with an implanted CIED treated in the University Hospital of Munich (LMU) between July 2012 and March 2024. Patients with non-MR conditionally labeled leads, abandoned or epicardial leads, as well as lead fragments, were included for analysis. Results: Across a total of 1010 MRIs (920 total MR-conditional device generators) performed in patients with an implanted CIED, there were no deaths, reports of discomfort, palpitations, heating, or ventricular arrythmias in the 24 h following MRI. Only 2/1010 MRIs were followed by a reported atrial arrhythmia within 24 h, both in patients with an MR-conditional pacemaker (PM) device without an abandoned lead. No significant changes in device function following MRI from baseline were observed across all included CIEDs. Lastly, no instances of severe malfunction, such as generator failure, loss of capture, electrical reset, or inappropriate inhibition of pacing, were found in post-MRI interrogation reports across all MRI studies. Conclusions: Based on the analysis of 1010 MRIs undergone by patients with CIEDs, following standardized device interrogation, manufacturer-advised device programming, monitoring of vital function, and manufacturer-advised reprogramming, MRI can be performed safely and without adverse events or changes in device function.

## 1. Introduction

Magnetic resonance imaging (MRI) represents the diagnostic image modality of choice in several conditions. With an increasing number of patients requiring MRI for diagnostic purposes, the issue of safety in patients with cardiac implantable electronic devices (CIED) undergoing this imaging modality will play an ever more important role. It is estimated that 50–75% of patients in the growing population of patients with cardiac devices will require MRI following implantation, though issues concerning static magnetic fields potentially exerting mechanical force on device components, pulsed radiofrequency (RF) field resulting in over-sensing, thermal damage at the tissue/electrode interface, and magnetic fields causing inappropriate pacing rates remain [1]. Despite concerns, most reports of serious adverse events following MRI were reported on legacy devices likely not in use today [2]. Further, despite the large number of MRIs required in patients with CIEDs, underutilization of this imaging modality has been reported in this population [3]. Also, the number of CIEDs implanted worldwide is likely to grow with an aging population [4]. In early guidelines, the diagnostic application of MRI was contraindicated in patients with cardiac devices [5]. Studies examining the safety of newer generation CIEDs labeled as MR-conditional have facilitated access to MRI for such patients [6]. Despite this, it is estimated that millions of patients with implanted devices do not meet the definition for MR-conditional devices, yet replacement of non-MR conditional generators and/or leads with MR-conditional devices may be associated with high complication rates that do not justify such procedures [7]. Further, evidence has shown the safety of MRI following the implantation of MR-conditional generators in patients with existing non-MR conditional leads, potentially avoiding the need for lead extraction [8].

For patients with non-MR-conditional PM systems, the most recent ESC guidelines provide a IIa recommendation for MRI in patients without and a IIb recommendation for patients with abandoned transvenous leads [9]. Despite existing concerns, a large prospective safety registry including 1500 non-MR-conditional CIEDs has shown that MRI does not lead to substantial changes in device settings or device failure [10]. Though smaller in size, prospective non-randomized real-world (non-MR-conditional and MR-conditional, thoracic, and non-thoracic images) data, including a reference group with MR-conditional devices undergoing imaging, found no increased adverse events [11]. Additionally, concerns exist pertaining to device-related artifacts due to ferromagnetic material in the field of view, particularly when imaging the cardiac/thoracic regions, despite evidence that MRI can provide high image quality and diagnostic value in patients with right-sided devices [12].

The purpose of this study was to assess the safety and device function following MRI in an unrestricted real-world cohort of patients with a wide array of cardiac devices. We hypothesized that irrespective of MR-conditional vs. non-MR-conditional labeling, MRI does not lead to an increased number of adverse events following standardized screening and identification of device status, the manufacturer-recommended programming, and peri-diagnostic monitoring of vital function with post-MRI reprogramming.

## 2. Methods

Study design and patient selection: In this current retrospective, single-center study, all patients undergoing CIED interrogation between July 2012 and March 2024 at the University Hospital of Munich (LMU) were screened for MRI. All patients with an MRI study and simultaneously implanted CIED were included for analysis. All data were extracted from patient-internal records reporting on device interrogations prior to and following MRI, with subsequent strict data anonymization. Prior to MRI, standardized identification of device labeling (MR-conditional vs. non-MR-conditional components), as well as absolute contraindications for MRI, were screened by a physician trained in electrophysiology and device interrogation. Patients > 18 years of age undergoing MRI with the following CIEDs were included for analysis: pacemaker, intracardiac cardioverter defibrillator (ICD), subcutaneous intracardiac cardioverter defibrillator (sICD), cardiac resynchronization therapy (CRT-P/D), or internal loop recorder (ILR). Patients with non-MR-conditional labeled leads, abandoned or epicardial leads, as well as lead fragments, were included for analysis. All clinical data were collected independently by two investigators. The validity and integrity of the clinical research dataset were controlled by one trained physician and one senior physician, as well as by our statistical team. Data collection and analysis were performed in accordance with the Declaration of Helsinki and German data protection laws. This study was approved by the local ethics committee (IRB numbers 17-662 and 20-641). This is the primary analysis of data that were exclusively compiled to investigate the safety of MRI in patients with CIEDs.

CIED interrogation: Prior to MRI, all devices were interrogated by a trained physician to assess for MR-conditional labeling of the device and lead combinations. All MR-conditional labeling was defined according to manufacturer protocol. Further, device constellations in which a single lead or generator of a CIED was labeled non-MR-conditional were adjudicated as non-MR-conditional. Detection of potentially high-risk scenarios such as non-MR-conditional labeling, presence of epicardial leads, abandoned leads, or fractured leads was noted in interrogation reports prior to imaging. A change in device programming to MRI mode according to manufacturer protocol was performed where available. In cases in which the ICD function was turned off, monitoring of vital function was performed according to hospital protocol. Following the MRI scan, all devices were interrogated and reprogrammed to baseline according to guideline recommendations [13]. All potential adverse events were reported in the post-MRI interrogation report. Lastly, all potential adverse events were reviewed and adjudicated as such by two senior investigators prior to statistical analysis.

MRI protocol: An MRI scan was performed according to institutional and manufacturer protocol in patients with MR-conditional CIED labeling. Patients were monitored using ECG, pulse oximetry, and verbal communication to maintain contact with MRI staff. All MRI imaging protocols were performed using a 1.5-T scanner.

Outcome variables: Primary outcome variables chosen prior to analysis included adverse events reported by device interrogations during and 24 h following MRI. Further outcomes included change in any parameter setting regarding battery status, lead sensing, lead pacing threshold, lead impedance, shock impedance for defibrillator leads, as well as essential device functions from baseline following MRI.

Statistical analysis: Statistical analysis was performed using R^®^ (version 4.0.3). Continuously distributed variables were reported as medians with interquartile ranges (25th and 75th percentile), and categorical variables were reported as absolute numbers and percentages. Patient characteristics were compared using Wilcoxon Rank-Sum tests for continuous variables and Fisher’s exact test or the Chi-square test for categorical variables. All tests were 2-tailed, and *p*-values < 0.05 were considered significant.

## 3. Results

Baseline characteristics: In this retrospective single-center study, a total of 1010 MRI scans undergone by patients with embedded CIEDs between July 2012 and March 2024 were included for analysis. Baseline characteristics of included patients are presented in Table 1. The median age of the study population was 74 years (62–80), and 66.5% of MRIs were performed in males. Most MRIs included for analysis were performed in patients with an implanted PM (604/1010), followed by ILRs (206/1010) and ICDs (110/1010). MR-conditional labeling according to the individual manufacturer for the individual device generators and leads is reported. A total of 91.1% (920/1010) of all MRIs were performed in patients with an implanted MR-conditional labeled generator. In most cases, right atrial (RA), right ventricular (RV), left ventricular (LV), and ICD leads were labeled as MR-conditional according to the manufacturer (91.0%, 89.4%, 88.2%, and 98.8%, respectively). MRI of the head was most frequently performed, with 53.8% of the total studies included (Table 2). For all devices included, an MRI was performed with a 1.5-T magnetic field.

Primary outcome: Across a total of 1010 MRIs performed in patients with an implanted CIED, there were no deaths, reports of discomfort, palpitations, heating, or ventricular arrythmias in the 24 h following MRI. Only 2/1010 MRIs were followed by an atrial arrhythmia within 24 h, both in patients with an MR-conditional PM device without an abandoned lead (Table 3).

Device function: Following the MRI scan, no significant change in the battery status of interrogated devices could be found in this cohort (Table 4). In all PM devices, battery status was labeled as “okay” prior to and following MRI (*n* = 1010). Further, for all PM devices included for analysis, there were no significant changes in pacing threshold [V], lead sensing [mV], or lead impedance from baseline across all right atrial and right ventricular leads. Further, there were no significant changes in the atrial (26.00% vs. 23.95%, *p* = 0.790) or ventricular (14.00% vs. 16.00%, *p* = 0.569) pacing rates in pre-MRI vs. post-MRI device interrogations across all PM devices. Similar results were found in patients undergoing MRI with an embedded ICD, with no significant change in pacing threshold, lead sensing, or lead impedance across the RA, RV, and ICD leads to baseline following MRI, respectively. Median ICD shock impedance [Ω] prior to MRI was 67.50 vs. 69.00 following MRI, *p* = 0.912. Additionally, CRT-(P/D) interrogation found no fluctuation of lead measurements and pacing percentages following MRI. In 206 MRIs performed in patients with an embedded ILR, no relevant change in battery status following imaging was reported. Lastly, no instances of severe malfunction, such as generator failure, loss of capture, electrical reset, or inappropriate inhibition of pacing/anti-tachycardia therapy were found in post-MRI interrogation reports across all MRI studies.

Abandoned and epicardial leads: Only 0.8% (8/1010) of all MRIs were performed with an abandoned lead, all of which were in patients with an implanted PM (Table 1). In patients with the identification of abandoned leads, the urgency of the required diagnostic MRI was weighed against the potential risks of device malfunction. In the eight MRIs performed with abandoned leads, 6/8 were performed for suspected stroke, one for suspected spinal cord compression, and one for a suspected pancreatic lesion. Only one epicardial lead was included for analysis. No patient with a lead fragment underwent an MRI in this cohort. There were no reported adverse events in patients with abandoned or epicardial leads.

## 4. Discussion

In this single-center retrospective study, an assessment of 1010 MRIs in patients with a wide array of cardiac devices for adverse events or significant changes in device function was performed. In this cohort, a total of two atrial arrythmias were reported within 24 h of MRI in post-MRI device interrogation reports, both in patients with an MR-conditional implanted PM. Slight alterations to device function following MRI were not statistically significant and not clinically relevant. Concerns of heating at the electrode-tissue boundary caused by RF of MRI and subsequent decline in pacing thresholds or even lead perforation were not observed, despite the inclusion of imaging studies with potentially increased risk such as MRI of the heart (8.1%) or breast (0.8%) [14]. Also, we included a large sample size with a wide variety of CIEDs from different manufacturers, a distinction to many similar studies assessing MRI safety and thus representing a strength of our data.

Martin et al. were the first to explore the safety of MRI at 1.5-T magnetic field strength in patients with CIEDs. Results encouraged further studies with increasingly less inclusion restriction of CIEDs for MRI safety assessment [15]. In an early study performed by Sommer et al., the authors showed the feasibility of pursuing safety analyses in non-pacing dependent patients undergoing extra-thoracic MRI under continuous monitoring, given device programming to asynchronous pacing mode was performed, despite remaining concerns of RF-related heating and subclinical myocardial necrosis [14]. This concern about an increase in myocardial necrosis markers has since been refuted by the lack of significant change following MRI in larger cohorts [16]. Further, the largest prospective registry to date exploring adverse events following non-cardiac MRI in patients with non-MR-conditional CIEDs included 1500 (1000 PMs, 500 ICDs) patients for safety analysis. Results were comparable to our cohort, as the authors found no deaths, lead failure, or loss of capture with only a few cases of atrial arrythmias in post-MRI device interrogation. As opposed to our study, patients undergoing cardiac MRI were excluded from the MagnaSafe registry, although subgroup analysis by region of MRI and MRI-conditional labeling was not performed in our analysis [10]. Though we did not perform a sub-analysis by MR-conditional vs. non-MR-conditional labeling, the small number of adverse events and non-significant change in device function in the total cohort do not lead us to assume differences in safety outcomes. This assumption is supported by evidence from a recent study comparing safety endpoints in 970 patients undergoing a total of 1148 MRI exams, in which no lead-related adverse clinical events or clinically significant immediate or late lead parameter changes could be observed following MRI in both MR-conditional and non-MR-conditional devices. Further, both thoracic and non-thoracic MRI scans were included in this analysis, with no signal for increased risk of adverse events following thoracic MRI [17]. While 53.8% of MRIs in our cohort were performed of the head vs. only 8% of the heart, we do not believe this to be a source of bias in our cohort, based on low safety events in studies both including and excluding thoracic MRI scans. It remains uncertain whether patients with CIEDs undergoing MRI at 3.0-T magnetic field strength are at higher risk for adverse events, and no guideline recommendations exist to guide clinical practice [9]. Safety in a total of 78 patients across four studies undergoing >1.5-T MRI with a CIED has been reported, with no serious adverse events or changes in device parameters [18,19,20,21]. These results are limited by the small sample size, and future studies should aim to include larger numbers of patients undergoing >1.5-T MRI with CIEDs. Further, the vast majority of generators and leads included in our cohort were labeled as MR-conditional, despite some difference between device types (i.e., 91.1% of overall generators labeled MR-conditional vs. 83.8% of CRT-D generators). Though not to be expected, we cannot exclude that the high rate of MR-conditional labeling in our cohort influenced the very low number of adverse events found following MRI. Concerns for higher risk of MRI-induced complications in generators implanted prior to 2001 have been raised in the past [22,23,24], though no devices of this age were included in our cohort.

According to the 2017 HRS expert consensus statement, the term MR-conditional refers to any device for which a specified MRI environment with specified conditions of non-hazardous use is achieved [13]. Thus, MR-conditional generators combined with non-MR-conditional leads or abandoned leads are deemed non-MR-conditional. Despite manufacturer pursuits to increase the number of available MR-conditional generators and leads, many patients with CIEDs not meeting these criteria will require MRI following device implantation. Solutions such as generator and/or lead exchange for MR-conditional components are not feasible and are associated with non-negligible risks [7]. Though awareness of technological advances and safety exists among clinicians surveyed on this topic, evidence showing that patients with non-MR-conditional CIEDs are more often denied access to MRI represents an inequity in current care [25]. MRI often represents the imaging modality of choice due to high tissue resolution; thus, efforts should be made to advance MRI safety in the growing population of patients with embedded cardiac devices and facilitate access to a larger number of patients. Such success is dependent on device manufacturers pursuing further testing of devices for approval of MR compatibility. Lastly, concerns about CIED-induced image artifacts have been raised. Assessment of the diagnostic value of the 82 included cardiac MRIs (8.1% of the total cohort) was not performed, though studies exist showing high quality and high diagnostic value of cardiac images in patients with right-sided devices [12]. Overall, our data provide a real-world experience of safe MRI imaging and low reported adverse event rates in patients with a wide array of CIEDs at a large university hospital. Our results add to the body of evidence showing that MRI in patients with MR-conditional and non-MR-conditional CIEDs is safe when proper identification of components, device programming, and monitoring is provided. While we provide a relatively large sample size, future works should follow a prospective multicenter design to better identify those patients at residual risk of adverse events. While such study designs may be challenging due to differences in institutional protocols, equipment, or trained personnel to program and reprogram CIEDs during MRI, they would provide a real-world experience that may reflect the difficulty of access to MRI for reasons other than safety concerns. These additional factors represent a possible source of bias and should be considered when interpreting safety outcomes. Lastly, future studies should implement standardized protocols for the assessment of MRI that follow guideline recommendations [9].

Limitations: The limitations inherent to this observational study mainly result from a lack of randomization and blinding. Although not to be expected due to the overall extremely low number of adverse events and non-significant change in device settings, differences in sub-analyses comparing MR-conditional with non-MR-conditional device constellations cannot be ruled out entirely. The lack of follow-up in our cohort represents a limitation, though most changes in device function are present shortly after imaging is performed. Additionally, the time from implantation to analysis of included leads and generators was quite short, potentially limiting the generalizability of our results. Lastly, CIED safety was not tested in 3-T MRI, and results should not be extrapolated for such imaging devices.

## 5. Conclusions

The present real-world study, based on the analysis of 1010 MRIs undergone by patients with CIEDs, showed that following standardized device interrogation, manufacturer-advised device programming, monitoring of vital function, and manufacturer-advised reprogramming, MRI could be performed safely and without adverse events or changes in device function. This should encourage future standardization of most cardiac devices to facilitate access to MRI for an increasing number of patients worldwide.

## Figures and Tables

**Table 1:** Baseline characteristics. CRT, cardiac resynchronization therapy; ICD, implantable cardioverter defibrillator; ILR, internal loop recorder; IQR, interquartile range; MRI, magnetic resonance imaging; *n*, number; N/A, not applicable; PM, pacemaker; RA, right atrium; RV, right ventricle; sICD, subcutaneous implantable cardioverter defibrillator. * Only one value is available.

Characteristics	Overall (*n* = 1010)	PM (*n* = 604)	ICD (*n* = 110)	sICD (*n* = 23)	CRT-P (*n* = 30)	CRT-D (*n* = 37)	ILR (*n* = 206)	*p*-Value
**Demographics**								
Age [years], median [IQR]	74.00 [62.00, 80.00]	77.00 [70.00, 82.00]	67.00 [58.00, 73.00]	45.00 [42.50, 47.00]	77.00 [75.25, 78.75]	70.00 [58.00, 80.00]	62.00 [55.00, 74.00]	<0.001
Sex at birth [male], *n* (%)	672 (66.5)	401 (66.4)	88 (80.0)	17 (73.9)	24 (80.0)	28 (75.7)	114 (55.3)	<0.001
**Aggregate**								
Manufacturer								
Boston Scientific, *n* (%)	78 (7.7)	33 (5.5)	11 (10.0)	23 (100.0)	3 (10.0)	9 (24.3)	0 (0.0)	<0.001
Medtronic, *n* (%)	558 (55.2)	302 (50.0)	44 (40.0)	0 (0.0)	13 (43.3)	12 (32.4)	187 (90.8)	<0.001
Biotronik, *n* (%)	239 (23.7)	186 (30.8)	22 (20.0)	0 (0.0)	4 (13.3)	11 (29.7)	15 (7.3)	<0.001
St. Jude Medical, *n* (%)	85 (8.4)	35 (5.8)	31 (28.2)	0 (0.0)	10 (33.3)	5 (13.5)	4 (1.9)	<0.001
MicroPort, *n* (%)	50 (5.0)	48 (7.9)	2 (1.8)	0 (0.0)	0 (0.0)	0 (0.0)	0 (0.0)	<0.001
Time since implantation [years], median [IQR]	1.75 [0.67, 3.58]	2.25 [0.75, 4.27]	2.00 [1.08, 4.27]	1.08 [0.62, 2.50]	1.62 [0.85, 2.58]	2.17 [1.08, 3.67]	1.00 [0.42, 1.92]	<0.001
Labeled as MR-conditional by manufacturer, *n* (%)	920 (91.1)	538 (89.1)	93 (84.5)	23 (100.0)	29 (96.7)	31 (83.8)	206 (100.0)	<0.001
**Right atrial lead**								
Right atrial lead, *n* (%)	643 (63.7)	535 (88.6)	43 (39.1)	N/A	29 (96.7)	36 (97.3)	N/A	<0.001
Manufacturer								
Boston Scientific RA lead, *n* (%)	40 (4.0)	23 (3.8)	7 (6.4)	N/A	3 (10.0)	7 (18.9)	N/A	<0.001
Medtronic RA lead, *n* (%)	313 (31.0)	279 (46.2)	10 (9.1)	N/A	13 (43.3)	11 (29.7)	N/A	<0.001
Biotronik RA lead, *n* (%)	190 (18.8)	166 (27.5)	8 (7.3)	N/A	3 (10.0)	13 (35.1)	N/A	0.025
St. Jude Medical RA lead, *n* (%)	61 (6.0)	29 (4.8)	18 (16.4)	N/A	9 (30.0)	5 (13.5)	N/A	<0.001
MicroPort RA lead, *n* (%)	24 (2.4)	24 (4.0)	0 (0.0)	N/A	0 (0.0)	0 (0.0)	N/A	0.309
Time since implantation [years], median [IQR]	2.50 [0.92, 4.77]	2.58 [0.92, 4.83]	2.00 [1.00, 3.25]	N/A	1.75 [0.92, 2.83]	2.54 [1.08, 4.35]	N/A	0.175
Labeled as MR-conditional by manufacturer, *n* (%)	585 (57.9)	485 (80.3)	41 (37.3)	N/A	27 (90.0)	32 (86.5)	N/A	0.157
**Right ventricular lead**								
Right ventricular lead, *n* (%)	630 (62.4)	599 (99.2)	1 (0.9)	N/A	30 (100.0)	0 (0.0)	N/A	<0.001
Manufacturer								
Boston Scientific RV lead, *n* (%)	38 (3.8)	35 (5.8)	0 (0.0)	N/A	3 (10.0)	0 (0.0)	N/A	0.451
Medtronic RV lead, *n* (%)	328 (32.5)	305 (50.5)	1 (0.9)	N/A	22 (73.3)	0 (0.0)	N/A	0.023
Biotronik RV lead, *n* (%)	188 (18.6)	184 (30.5)	0 (0.0)	N/A	4 (13.3)	0 (0.0)	N/A	0.075
St. Jude Medical RV lead, *n* (%)	33 (3.3)	33 (5.5)	0 (0.0)	N/A	0 (0.0)	0 (0.0)	N/A	0.426
MicroPort RV lead, *n* (%)	34 (3.4)	34 (5.6)	0 (0.0)	N/A	0 (0.0)	0 (0.0)	N/A	0.430
Time since implantation [years], median [IQR]	2.42 [0.75, 4.75]	2.42 [0.75, 4.83]	21.92 [21.92, 21.92] *	N/A	1.79 [0.92, 2.77]	0.58 [0.58, 0.58] *	N/A	0.110
Labeled as MR-conditional by manufacturer, *n* (%)	563 (55.7)	536 (88.7)	0 (0.0)	N/A	27 (90.0)	0 (0.0)	N/A	0.084
**Left ventricular lead**								
Left ventricular lead, *n* (%)	68 (6.7)	2 (0.3)	0 (0.0)	N/A	30 (100.0)	36 (97.3)	N/A	<0.001
Manufacturer								
Boston Scientific LV lead, *n* (%)	12 (1.2)	0 (0.0)	0 (0.0)	N/A	4 (13.3)	8 (21.6)	N/A	0.678
Medtronic LV lead, *n* (%)	24 (2.4)	2 (0.3)	0 (0.0)	N/A	11 (36.7)	11 (29.7)	N/A	0.159
Biotronik LV lead, *n* (%)	15 (1.5)	0 (0.0)	0 (0.0)	N/A	4 (13.3)	11 (29.7)	N/A	0.211
St. Jude Medical LV lead, *n* (%)	16 (1.6)	0 (0.0)	0 (0.0)	N/A	10 (33.3)	6 (16.2)	N/A	0.231
MicroPort LV lead, *n* (%)	0 (0.0)	0 (0.0)	0 (0.0)	N/A	0 (0.0)	0 (0.0)	N/A	-
Time since implantation [years], median [IQR]	2.12 [0.98, 3.52]	1.71 [1.23, 2.19]	-	N/A	1.62 [0.85, 2.58]	2.54 [1.33, 4.02]	N/A	0.085
Labeled as MR-conditional by manufacturer, *n* (%)	60 (5.9)	2 (0.3)	0 (0.0)	N/A	27 (90.0)	31 (83.8)	N/A	0.464
**Implantable cardioverter defibrillator lead**								
Implantable cardioverter defibrillator lead, *n* (%)	169 (16.7)	N/A	109 (99.1)	23 (100.0)	N/A	37 (100.0)	N/A	<0.001
Manufacturer								
Boston Scientific ICD lead, *n* (%)	45 (4.5)	N/A	14 (12.7)	22 (95.7)	N/A	9 (24.3)	N/A	<0.001
Medtronic ICD lead, *n* (%)	49 (4.9)	N/A	38 (34.5)	0 (0.0)	N/A	11 (29.7)	N/A	0.001
Biotronik ICD lead, *n* (%)	37 (3.7)	N/A	24 (21.8)	1 (4.3)	N/A	12 (32.4)	N/A	0.029
St. Jude Medical ICD lead, *n* (%)	37 (3.7)	N/A	32 (29.1)	0 (0.0)	N/A	5 (13.5)	N/A	0.001
MicroPort ICD lead, *n* (%)	1 (0.1)	N/A	1 (0.9)	0 (0.0)	N/A	0 (0.0)	N/A	>0.999
Time since implantation [years], median [IQR]	2.00 [1.00, 4.08]	N/A	2.00 [1.08, 5.08]	1.08 [0.62, 2.50]	N/A	2.50 [1.08, 3.83]	N/A	0.049
Labeled as MR-conditional by manufacturer, *n* (%)	167 (16.5)	N/A	107 (97.3)	23 (100.0)	N/A	37 (100.0)	N/A	>0.999
**Abandoned leads**								
Abandoned leads, *n* (%)	8 (0.8)	8 (1.3)	0 (0.0)	0 (0.0)	0 (0.0)	0 (0.0)	0 (0.0)	0.541
Number of abandoned leads per patient								
One, *n* (%)	8 (0.8)	8 (1.3)	0 (0.0)	0 (0.0)	0 (0.0)	0 (0.0)	0 (0.0)	0.541
Manufacturer								
Medtronic, *n* (%)	2 (0.2)	2 (0.3)	0 (0.0)	0 (0.0)	0 (0.0)	0 (0.0)	0 (0.0)	>0.999
Biotronik, *n* (%)	4 (0.4)	4 (0.7)	0 (0.0)	0 (0.0)	0 (0.0)	0 (0.0)	0 (0.0)	>0.999
St. Jude Medical, *n* (%)	2 (0.2)	2 (0.3)	0 (0.0)	0 (0.0)	0 (0.0)	0 (0.0)	0 (0.0)	>0.999
Location								
RA, *n* (%)	5 (0.5)	5 (0.8)	0 (0.0)	0 (0.0)	0 (0.0)	0 (0.0)	0 (0.0)	>0.999
RV, *n* (%)	3 (0.3)	3 (0.5)	0 (0.0)	0 (0.0)	0 (0.0)	0 (0.0)	0 (0.0)	>0.999
Time since implantation [years], median [IQR]	9.88 [6.98, 12.50]	9.88 [6.98, 12.50]	-	-	-	-	-	-
**Epicardial leads**								
Epicardial leads, *n* (%)	1 (0.1)	0 (0.0)	0 (0.0)	0 (0.0)	0 (0.0)	1 (2.7)	0 (0.0)	0.089
Number of epicardial leads per patient								
One, *n* (%)	1 (0.1)	0 (0.0)	0 (0.0)	0 (0.0)	0 (0.0)	1 (2.7)	0 (0.0)	0.089
Manufacturer								
Medtronic, *n* (%)	1 (0.1)	0 (0.0)	0 (0.0)	0 (0.0)	0 (0.0)	0 (0.0)	0 (0.0)	-
Location								
Sinus coronarius, *n* (%)	1 (0.1)	0 (0.0)	0 (0.0)	0 (0.0)	0 (0.0)	1 (2.7)	0 (0.0)	0.089
Time since implantation [years], median [IQR]	0.08 [0.08, 0.08] *	-	-	-	-	-	-	-
**Lead fragment**								
Lead fragment, *n* (%)	0 (0.0)	0 (0.0)	0 (0.0)	0 (0.0)	0 (0.0)	0 (0.0)	0 (0.0)	-

**Table 2:** MRI attributes. CRT, cardiac resynchronization therapy; ICD, implantable cardioverter defibrillator; ILR, internal loop recorder; IQR, interquartile range; MRI, magnetic resonance imaging; *n*, number; PM, pacemaker; RA, right atrium; RV, right ventricle; sICD, subcutaneous implantable cardioverter defibrillator. For all devices included, an MRI was performed with a 1.5-T magnetic field.

Characteristics	Overall (*n* = 1010)	PM (*n* = 604)	ICD (*n* = 110)	sICD (*n* = 23)	CRT-P (*n* = 30)	CRT-D (*n* = 37)	ILR (*n* = 206)	*p*-Value
**MRI attributes**								
Anatomic region imaged								
Head, *n* (%)	543 (53.8)	324 (53.6)	74 (67.3)	7 (30.4)	15 (50.0)	10 (27.0)	113 (54.9)	<0.001
Spine, *n* (%)	115 (11.4)	79 (13.1)	9 (8.2)	2 (8.7)	3 (10.0)	8 (21.6)	14 (6.8)	0.046
Head and spine, *n* (%)	24 (2.4)	17 (2.8)	2 (1.8)	0 (0.0)	0 (0.0)	3 (8.1)	2 (1.0)	0.189
Head and neck, *n* (%)	3 (0.3)	3 (0.5)	0 (0.0)	0 (0.0)	0 (0.0)	0 (0.0)	0 (0.0)	0.781
Neck, *n* (%)	3 (0.3)	2 (0.3)	0 (0.0)	0 (0.0)	0 (0.0)	0 (0.0)	1 (0.5)	1.000
Neck and thorax, *n* (%)	1 (0.1)	1 (0.2)	0 (0.0)	0 (0.0)	0 (0.0)	0 (0.0)	0 (0.0)	>0.999
Upper extremity, *n* (%)	16 (1.6)	7 (1.2)	0 (0.0)	2 (8.7)	0 (0.0)	2 (5.4)	5 (2.4)	0.022
Upper extremity and spine, *n* (%)	1 (0.1)	1 (0.2)	0 (0.0)	0 (0.0)	0 (0.0)	0 (0.0)	0 (0.0)	>0.999
Thorax, *n* (%)	4 (0.4)	3 (0.5)	0 (0.0)	0 (0.0)	0 (0.0)	1 (2.7)	0 (0.0)	0.355
Thorax and abdomen, *n* (%)	1 (0.1)	1 (0.2)	0 (0.0)	0 (0.0)	0 (0.0)	0 (0.0)	0 (0.0)	>0.999
Thorax and spine, *n* (%)	1 (0.1)	1 (0.2)	0 (0.0)	0 (0.0)	0 (0.0)	0 (0.0)	0 (0.0)	>0.999
Breast, *n* (%)	8 (0.8)	8 (1.3)	0 (0.0)	0 (0.0)	0 (0.0)	0 (0.0)	0 (0.0)	0.541
Heart, *n* (%)	82 (8.1)	23 (3.8)	11 (10.0)	7 (30.4)	3 (10.0)	6 (16.2)	32 (15.5)	<0.001
Heart and thorax, *n* (%)	1 (0.1)	0 (0.0)	0 (0.0)	0 (0.0)	0 (0.0)	0 (0.0)	1 (0.5)	0.402
Heart and prostate, *n* (%)	1 (0.1)	0 (0.0)	0 (0.0)	0 (0.0)	0 (0.0)	1 (2.7)	0 (0.0)	0.089
Abdomen, *n* (%)	95 (9.4)	67 (11.1)	1 (0.9)	0 (0.0)	2 (6.7)	2 (5.4)	23 (11.2)	0.002
Abdomen and lower extremity, *n* (%)	1 (0.1)	1 (0.2)	0 (0.0)	0 (0.0)	0 (0.0)	0 (0.0)	0 (0.0)	>0.999
Rectum, *n* (%)	1 (0.1)	0 (0.0)	1 (0.9)	0 (0.0)	0 (0.0)	0 (0.0)	0 (0.0)	0.198
Prostate, *n* (%)	25 (2.5)	13 (2.2)	5 (4.5)	1 (4.3)	0 (0.0)	0 (0.0)	6 (2.9)	0.483
Lower extremity, *n* (%)	74 (7.3)	45 (7.5)	7 (6.4)	4 (17.4)	7 (23.3)	4 (10.8)	7 (3.4)	0.002
Lower extremity and spine, *n* (%)	3 (0.3)	2 (0.3)	0 (0.0)	0 (0.0)	0 (0.0)	0 (0.0)	1 (0.5)	>0.999
Lower extremity and prostate, *n* (%)	2 (0.2)	2 (0.3)	0 (0.0)	0 (0.0)	0 (0.0)	0 (0.0)	0 (0.0)	>0.999
Lower extremity, spine and head, *n* (%)	1 (0.1)	1 (0.2)	0 (0.0)	0 (0.0)	0 (0.0)	0 (0.0)	0 (0.0)	>0.999
Whole body, *n* (%)	2 (0.2)	2 (0.3)	0 (0.0)	0 (0.0)	0 (0.0)	0 (0.0)	0 (0.0)	>0.999

**Table 3:** Related adverse events during and 24h post-MRI. CRT, cardiac resynchronization therapy; ICD, implantable cardioverter defibrillator; ILR, internal loop recorder; IQR, interquartile range; MRI, magnetic resonance imaging; *n*, number; PM, pacemaker; sICD, subcutaneous implantable cardioverter defibrillator. Two episodes of atrial arrhythmia were reported within 24 h of MRI in two patients with a PM device. No further adverse events were reported in the cohort.

Characteristics	Overall (*n* = 1010)	PM (*n* = 604)	ICD (*n* = 110)	sICD (*n* = 23)	CRT-P (*n* = 30)	CRT-D (*n* = 37)	ILR (*n* = 206)	*p*-Value
**Related adverse events during and 24h post MRI in patients without abandoned and epicardial leads or lead fragments**								
Discomfort, *n* (%)	0 (0.0)	0 (0.0)	0 (0.0)	0 (0.0)	0 (0.0)	0 (0.0)	0 (0.0)	-
Palpitation, *n* (%)	0 (0.0)	0 (0.0)	0 (0.0)	0 (0.0)	0 (0.0)	0 (0.0)	0 (0.0)	-
Heating, *n* (%)	0 (0.0)	0 (0.0)	0 (0.0)	0 (0.0)	0 (0.0)	0 (0.0)	0 (0.0)	-
Sensation of device migration, *n* (%)	0 (0.0)	0 (0.0)	0 (0.0)	0 (0.0)	0 (0.0)	0 (0.0)	0 (0.0)	-
Abnormal vital signs, *n* (%)	0 (0.0)	0 (0.0)	0 (0.0)	0 (0.0)	0 (0.0)	0 (0.0)	0 (0.0)	-
**Atrial arrhythmia, *n* (%)**	2 (0.2)	2 (0.3)	0 (0.0)	0 (0.0)	0 (0.0)	0 (0.0)	0 (0.0)	>0.999
Ventricular arrhythmia, *n* (%)	0 (0.0)	0 (0.0)	0 (0.0)	0 (0.0)	0 (0.0)	0 (0.0)	0 (0.0)	-
Death, *n* (%)	0 (0.0)	0 (0.0)	0 (0.0)	0 (0.0)	0 (0.0)	0 (0.0)	0 (0.0)	-

**Table 4:** Device function pre vs. post-MRI. CRT, cardiac resynchronization therapy; ICD, implantable cardioverter defibrillator; ILR, internal loop recorder; IQR, interquartile range; MRI, magnetic resonance imaging; *n*, number; PM, pacemaker; RA, right atrial; RV, right ventricular; sICD, subcutaneous implantable cardioverter defibrillator; V, volt; mV, millivolt.

PM	Pre MRI	Post MRI	*p*-Value
**Battery**			
Battery level okay, *n* (%)	604 (100.0)	604 (100.0)	-
**Right atrial lead**			
RA lead Pacing (threshold) [V], median [IQR]	0.75 [0.50, 0.80]	0.70 [0.50, 0.80]	0.290
RA lead Sensing [mV], median [IQR]	3.00 [1.90, 4.20]	3.00 [1.80, 4.57]	0.817
RA lead Impedance [Ω], median [IQR]	456.00 [399.00, 526.00]	447.00 [399.00, 507.00]	0.411
**Right ventricular lead**			
RV lead Pacing (threshold) [V], median [IQR]	0.75 [0.62, 1.00]	0.75 [0.62, 1.00]	0.899
RV lead Sensing [mV], median [IQR]	10.80 [8.00, 13.70]	10.80 [8.10, 13.90]	0.779
RV lead Impedance [Ω], median [IQR]	525.00 [456.00, 589.00]	513.00 [456.00, 589.00]	0.340
**Atrial pacing**			
Atrial pacing rate [%], median [IQR]	26.00 [2.60, 70.00]	23.95 [1.55, 74.25]	0.790
**Ventricular pacing**			
Ventricular pacing rate [%], median [IQR]	14.00 [0.26, 93.00]	16.00 [0.20, 97.00]	0.569
**ICD**	**Pre MRI**	**Post MRI**	***p*-value**
**Battery**			
Battery level okay, *n* (%)	110 (100.0)	110 (100.0)	-
**Right atrial lead**			
RA lead Pacing (threshold) [V], median [IQR]	0.80 [0.62, 1.25]	0.80 [0.58, 1.25]	0.842
RA lead Sensing [mV], median [IQR]	2.35 [1.60, 4.02]	2.15 [1.52, 4.35]	0.830
RA lead Impedance [Ω], median [IQR]	446.50 [388.00, 516.30]	453.00 [388.00, 523.10]	0.667
**Right ventricular lead**			
RV lead Pacing (threshold) [V], median [IQR]	0.62 [0.60, 1.20]	0.90 [0.60, 1.32]	>0.999
RV lead Sensing [mV], median [IQR]	11.70 [9.40, 17.20]	11.70 [11.30, 17.20]	>0.999
RV lead Impedance [Ω], median [IQR]	478.00 [475.00, 487.00]	478.00 [475.00, 488.00]	>0.999
**ICD lead**			
ICD lead Pacing (threshold) [V], median [IQR]	0.75 [0.60, 1.00]	0.80 [0.60, 1.00]	0.606
ICD lead Sensing [mV], median [IQR]	11.50 [8.40, 13.97]	11.50 [8.40, 13.57]	0.946
ICD lead Impedance [Ω], median [IQR]	456.00 [403.50, 538.00]	459.00 [403.00, 538.00]	0.917
**Implantable cardioverter defibrillator lead**			
Shock Impedance [Ω], median [IQR]	67.50 [59.00, 77.75]	69.00 [59.00, 77.00]	0.912
**Atrial pacing**			
Atrial pacing rate [%], median [IQR]	2.35 [0.00, 33.75]	1.00 [0.00, 35.00]	0.881
**Ventricular pacing**			
Ventricular pacing rate [%], median [IQR]	0.00 [0.00, 0.91]	0.00 [0.00, 0.30]	0.585
**sICD**	**Pre MRI**	**Post MRI**	***p*-value**
**Battery**			
Battery level okay, *n* (%)	23 (100.0)	23 (100.0)	-
**Implantable cardioverter defibrillator lead**			
Shock Impedance okay, *n* (%)	5 (21.7)	5 (21.7)	-
**CRT-P**	**Pre MRI**	**Post MRI**	***p*-value**
**Battery**			
Battery level okay, *n* (%)	30 (100.0)	30 (100.0)	-
**Right atrial lead**			
RA lead Pacing (threshold) [V], median [IQR]	0.75 [0.50, 1.00]	0.75 [0.50, 1.00]	0.850
RA lead Sensing [mV], median [IQR]	1.90 [0.90, 3.10]	2.30 [1.00, 3.40]	0.575
RA lead Impedance [Ω], median [IQR]	418.00 [399.00, 450.00]	418.00 [380.00, 450.00]	0.639
**Right ventricular lead**			
RV lead Pacing (threshold) [V], median [IQR]	0.70 [0.50, 0.75]	0.55 [0.50, 0.75]	0.906
RV lead Sensing [mV], median [IQR]	12.00 [11.70, 13.20]	12.00 [11.65, 13.05]	0.931
RV lead Impedance [Ω], median [IQR]	563.00 [498.75, 592.75]	563.00 [487.00, 589.00]	0.749
**Left ventricular lead**			
LV lead Pacing (threshold) [V], median [IQR]	1.65 [1.02, 1.75]	1.50 [1.00, 2.00]	0.837
LV lead Sensing [mV], median [IQR]	11.45 [9.45, 14.07]	9.10 [8.85, 13.20]	>0.999
LV lead Impedance [Ω], median [IQR]	813.50 [556.25, 966.00]	813.50 [538.25, 966.00]	0.961
**Atrial pacing**			
Atrial pacing rate [%], median [IQR]	9.10 [0.90, 75.00]	9.10 [0.90, 75.00]	0.907
**Ventricular pacing**			
Ventricular pacing rate [%], median [IQR]	94.00 [91.00, 97.70]	95.00 [91.40, 99.00]	0.421
**CRT-D**	**Pre MRI**	**Post MRI**	***p*-value**
**Battery**			
Battery level okay, *n* (%)	37 (100.0)	37 (100.0)	-
**Right atrial lead**			
RA lead Pacing (threshold) [V], median [IQR]	0.75 [0.68, 0.83]	0.75 [0.70, 0.90]	0.616
RA lead Sensing [mV], median [IQR]	2.50 [1.50, 5.30]	2.40 [1.50, 4.92]	0.758
RA lead Impedance [Ω], median [IQR]	504.00 [394.25, 565.00]	505.00 [399.00, 565.00]	0.995
**Right ventricular lead**			
RV lead Pacing (threshold) [V], median [IQR] *	0.60 [0.60, 0.60]	0.60 [0.60, 0.60]	-
RV lead Sensing [mV], median [IQR] *	23.40 [23.40, 23.40]	23.40 [23.40, 23.40]	-
RV lead Impedance [Ω], median [IQR] *	496.00 [496.00, 496.00]	496.00 [496.00, 496.00]	-
* only one value available			
**Left ventricular lead**			
LV lead Pacing (threshold) [V], median [IQR]	1.00 [0.74, 1.32]	1.00 [0.75, 1.50]	0.950
LV lead Sensing [mV], median [IQR]	17.90 [13.95, 21.20]	8.80 [8.75, 16.65]	0.507
LV lead Impedance [Ω], median [IQR]	559.00 [410.00, 670.00]	560.00 [381.00, 665.00]	0.841
**Implantable cardioverter defibrillator lead**			
Shock Impedance [Ω], median [IQR]	62.00 [56.00, 81.50]	62.00 [56.50, 79.50]	0.888
**Atrial pacing**			
Atrial pacing rate [%], median [IQR]	26.00 [1.00, 72.50]	26.00 [1.00, 72.50]	0.883
**Ventricular pacing**			
Ventricular pacing rate [%], median [IQR]	99.00 [95.10, 99.80]	99.00 [96.10, 99.80]	0.857
**ILR**	**Pre MRI**	**Post MRI**	***p*-value**
**Battery**			
Battery level okay, *n* (%)	206 (100.0)	206 (100.0)	-

## Data Availability

The datasets analyzed during the current study are available from the corresponding author upon reasonable request. The data are not publicly available due to ethical restrictions and legal constraints. Readers may contact Enzo Lüsebrink for reasonable requests for the data. De-identified data may be provided after approval from the ethical review board.
